# Regulatory effects of electronic beam irradiation on mir-21/smad7-mediated collagen I synthesis in keloid-derived fibroblasts

**DOI:** 10.1242/bio.018770

**Published:** 2016-09-30

**Authors:** Shifeng Li, Wei Liu, Ying Lei, Jianhong Long

**Affiliations:** 1 Department of Plastic and Aesthetic Surgery, Xiangya Hospital of Central South University, Changsha City, Hunan Province 410008, China; 2 Department of Plastic and Cosmetic Surgery, The first people's hospital of Chenzhou, Chenzhou City, Hunan Province 423000, China; 3 Department of Pathology, The Affiliated Cancer Hospital of Xiangya Medical School, Changsha City, Hunan Province 410013, China; 4 Department of Plastic and Cosmetic Surgery, The people's hospital of Hunan province, Changsha City, Hunan Province 410000, China

**Keywords:** Electronic beam irradiation, Radiotherapy, Keloid scarring, mir-21, smad7, p38 phosphorylation

## Abstract

Keloid scarring is an abnormal pathological scar characterized by excessive fibro proliferation and extracellular matrix deposition. Electronic beam irradiation is commonly used with surgical removal to control high recurrence rates of keloid scarring; however, the mechanism remains unknown. In this study, we used keloid-derived primary fibroblasts (KF) as the cell model, and a dose of 15 Gy energy, followed by quantitative PCR (qPCR), western blotting and gene overexpression/knock down techniques were used to reveal the molecular mechanisms affected by electronic beam irradiation. We found that mir-21 was highly expressed in KF and was downregulated by irradiation. We also showed that smad7 was a direct target of mir-21. Moreover, the expression level of smad7 was low in KF and upregulated by irradiation. We also found that smad7 controls Col-1 synthesis by mediating p38 phosphorylation, and this process was affected by electronic beam irradiation. The regulatory effect of electronic beam irradiation on the expression of mir-21, smad7, p38, p-p38 and Col-1 could be partly restored by mir-21 overexpression achieved by mir-21 mimic transfection. In conclusion, our data demonstrated that mir-21/smad7 regulated Col-1 expression in KF and that electronic beam irradiation was capable of decreasing Col-1 production by modifying mir-21/smad7-mediated p38 activation. This is the first report identifying the effects of electronic beam irradiation on miRNAs, providing a novel strategy to discover the molecular mechanisms of radiotherapy.

## Abstract (precis)

**Summary:** Using primary keloid-derived fibroblasts, we demonstrate that electronic beam irradiation inhibits the recurrence of keloid scarring by suppressing collagen I expression via mir-21/smad7-mediated p38 activation.

## INTRODUCTION

Keloid scarring, known as keloid disease, is an abnormal pathological scar that aggressively grows beyond the boundary of the original wound and invades surrounding healthy skin, which leads to itching, pain, and a stretching sensation (Ogawa et al., 2007; Shih and Bayat, 2010). Although surgical excision is effective, high recurrence rates, ranging from 55% to 100%, can cause an even more severe situation in patients (Butler et al., 2008); however, as previously reported, postoperative electronic beam irradiation is one of the most effective treatments for the prevention of recurrent keloids (Ogawa et al., 2007; Stadelmann et al., 1998).

Currently, the TGF-β pathway has been confirmed to be involved in excessive proliferation of fibroblasts and collagen accumulation during keloid pathogenesis (Seifert and Mrowietz, 2009; Shih and Bayat, 2010). Smad7, a key negative regulatory smad, was reported to be downregulated in keloids, as well as other fibrotic tissues, such as the lung, liver and kidney (Briones-Orta et al., 2011). Overproduction of collagen resulted from low expression of smad7 or other smad-independent signaling pathways (Yu et al., 2006). Although electronic beam irradiation could control collagen synthesis, the molecular mechanisms are still unknown (Stadelmann et al., 1998). P38 MAPK regulates matrix metalloproteinase (MMP) gene expression to create a balance in the extracellular matrix accumulation for normal skin that is highly activated during keloid scarring (Lam et al., 2005). Evidence has also shown that crosstalk exists between the TGF-β and p38 signaling pathways, and smad7 may play an important role in this process (Edlund et al., 2003; Iwai et al., 2008).

Since their discovery, miRNAs were demonstrated to be involved in cell proliferation, differentiation, development, metabolism, apoptosis, and other physiological activities (Carthew, 2006). Increasingly more miRNAs, such as mir-199a (Wu et al., 2014), mir-196a (Kashiyama et al., 2012), mir-29, let-7 (Suh et al., 2012) and mir-21 (Zhu et al., 2014), were found to be functional in fibroblast proliferation and extracellular matrix accumulation. Furthermore, mir-21 regulates the TGF-β pathway by directly targeting smad7 3′UTR (Li et al., 2013).

In summary, there may be a relationship between mir-21/smad7, p38 activation and collagen synthesis. In this research, we used primary keloid-derived fibroblasts (KF) obtained from patients as the cell model. We also used qPCR, western blotting and miRNA modifications to verify the existence of the previously mentioned network and how electronic beam irradiation affected this process in controlling collagen synthesis.

## RESULTS

### Collagen I was highly expressed in keloid-derived fibroblasts compared with normal skin fibroblasts

**Fig. 1.**
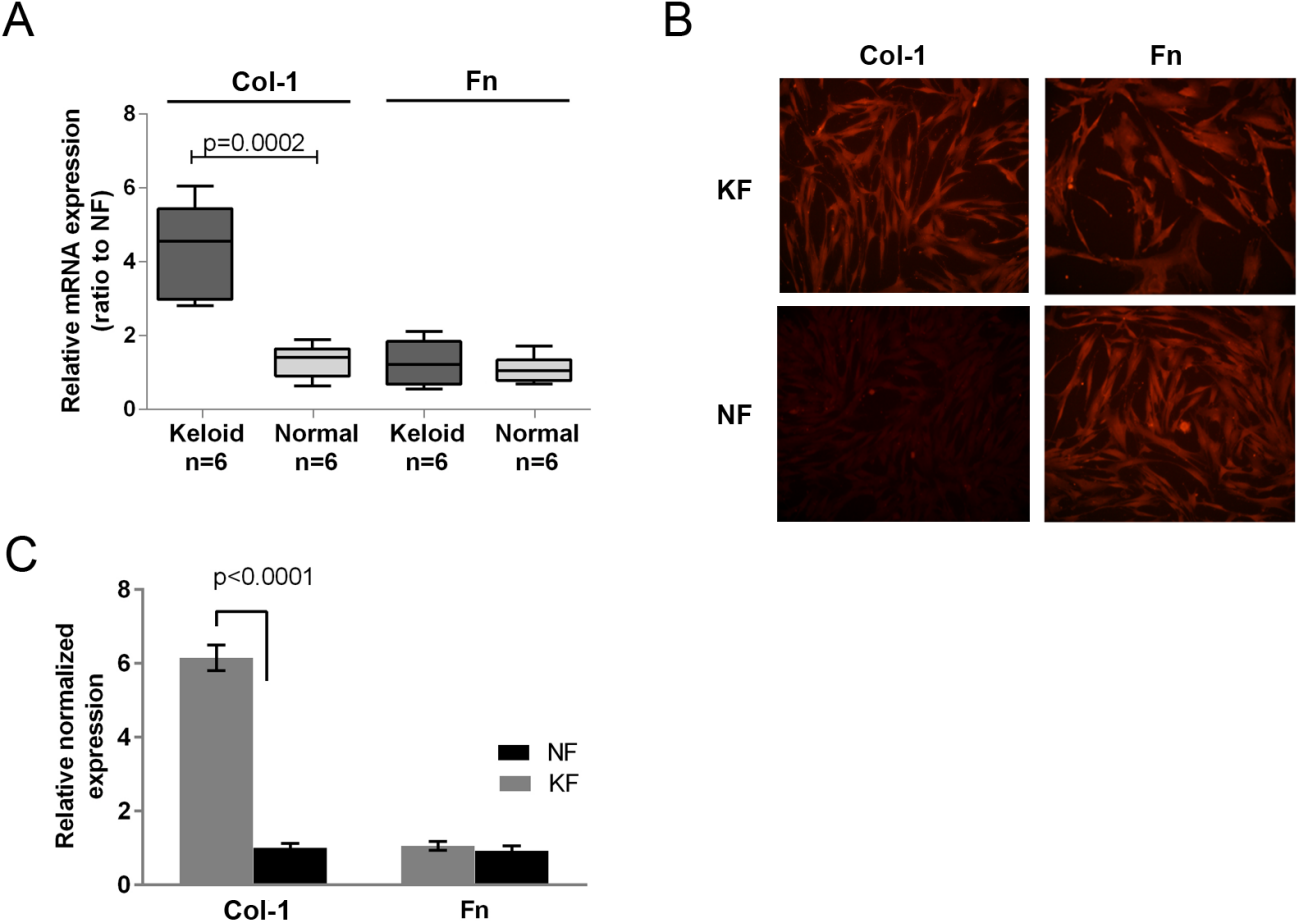
**Characterization of keloid-derived fibroblasts (KF) and normal skin fibroblasts (NF) with immunofluorescence staining and quantitative PCR.** (A) qPCR results demonstrated Col-1 was expressed higher in keloid samples than normal skin samples, while fibronectin (Fn) expression was similar between keloid and normal skin samples. Box and whisker plot show the minimum value, second quartile, median value, third quartile and maximum value of Col-1 or Fn mRNA expression in each group. (B) Immunofluorescence images indicated the expression of Col-1 was higher in KF, while no significant differences for Fn was found in expression between KF and NF. (C) Q-PCR results demonstrated that the expression tends of Col-1 and Fn were coincident with immunofluorescence results. Figure is representative of three experiments with similar results (normalized to NF group). Error bar indicates standard deviation (s.d.).

Primary KF and normal skin fibroblasts (NF) were isolated from keloid and normal skin tissues from the same patient. After isolation, the cells were maintained in our laboratory for three passages before characterizing the expression levels of Col-1 and Fn with immunofluorescence staining and qPCR. As shown in Fig. 1A, in keloid skin tissue, mRNA level of Col-1 was higher than that of the normal skin tissue, while the mRNA level of Fn was similar between keloid and normal skin samples. Similar to the tissue samples, in isolated cells (Fig. 1B,C), both KF and NF expressed Fn, and there were no significant differences in the expression levels. However, compared with expression levels in NF, Col-1 was highly expressed in KF, which was consistent with the previous reports (Arakawa et al., 1990; Shih and Bayat, 2010).

### Electron beam irradiation had negative effects on the proliferation rate and migration capacity of keloid-derived fibroblasts and Col-1 expression in keloid-derived fibroblasts

**Fig. 2.**
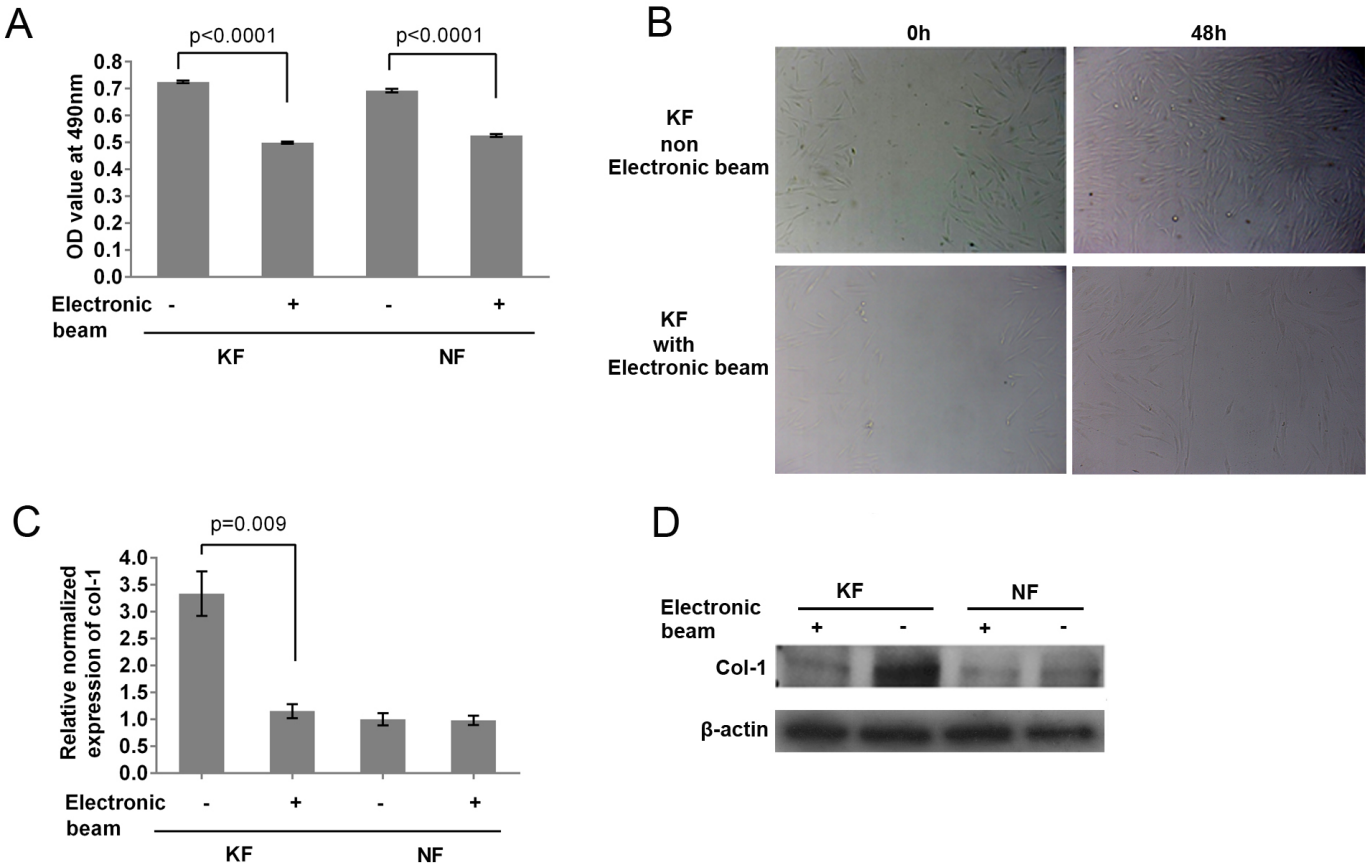
**Electron beam irradiation had negative effects on the proliferation rate and migration capacity of keloid-derived fibroblasts and Col-1 expression in keloid-derived fibroblasts.** (A) MTT assay showed cell growth for both KF and NF was decreased by electron beam irradiation. The MTT assay was performed 72 h after electron beam irradiation. (B) The wound healing assay showed that the cell migration capacity was damaged by electron beam irradiation. The wound healing assay started on the day of electron beam irradiation. Images were taken on the first day and 48 h later. (C) qPCR data indicated that electron beam irradiation suppressed the expression of Col-1 in KF rather than in NF. (D) Western blotting data indicated that electron beam irradiation suppressed the expression of Col-1. Figure is representative of three experiments with similar results (normalized to NF group). Error bars in A and C indicate s.d.

Electron beam irradiation is currently used with surgery on scar treatment to decrease the recurrence of scars. In this study, we irradiated KF and NF seeded in 6-well plates with a concentration of 5×10^5^ cells per well with the dose of 15 Gy at 8 Mev energy. After irradiation, we measured cell proliferation with the MTT assay at the 72 h time point. Fig. 2A indicated that electron beam irradiation slowed down cell proliferation rates of KF by 32% and NF by 21%, compared with the non-irradiated group. We also compared the migration capacity of KF in the irradiated and non-irradiated group via the wound-healing test. As indicated in Fig. 2B, KF in the non-irradiated group migrated into the wound area and nearly covered the whole scratch after 48 h, while cells in the irradiated group did not migrate as far. Therefore, electron beam irradiation also damaged cell migration capacity. Because Col-1 was highly expressed in KF, we determined whether electron beam irradiation suppressed the expression level of Col-1 with qPCR and western blotting. As shown in Fig. 2C,D, both mRNA and protein levels of Col-1 in KF were downregulated after irradiation. Interestingly, electron beam irradiation did not change the expression of Col-1 in NF at the mRNA or protein levels. Therefore, electron beam irradiation might modulate the expression of Col-1 to exhibit its therapeutic effects on the scar.

### Electron beam irradiation decreased p38 phosphorylation and modulated mir-21/smad7 signaling

**Fig. 3.**
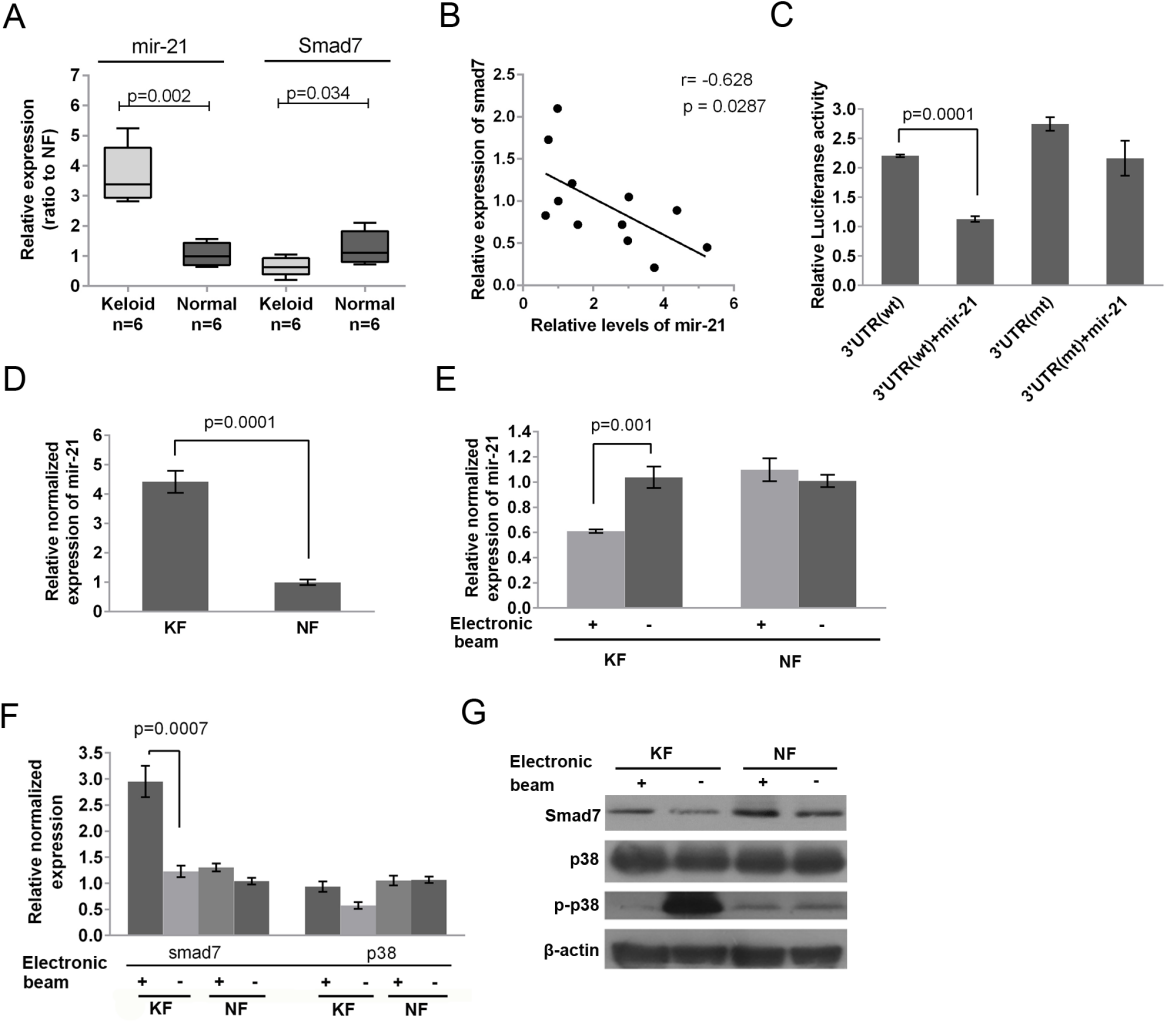
**Electron beam irradiation decreased p38 phosphorylation and modulated mir-21/smad7 signaling.** (A) qPCR results demonstrated the level of mir-21 was higher in keloid samples than normal skin samples, while the expression of smad7 was lower in keloid samples than normal skin samples. (B) A negative correlation between smad7 mRNA and mir-21 expression levels was observed. (C) Luciferase assay results revealed that mir-21 directly downregulated the expression of smad7 by binding to its 3′UTR. (D) Quantitative PCR data indicated that mir-21 levels were higher in KF compared with NF. KF and NF samples without electron beam irradiation were collected for this test. (E) qPCR data indicated that electron beam irradiation suppressed the expression of mir-21 in KF compared with NF. (F) qPCR data indicated that electron beam irradiation increased the mRNA level of smad7 in KF compared with NF, though it did not significantly affect the expression of p38. (G) Western blotting data indicated that electron beam irradiation increased the expression of smad7 and suppressed the phosphorylation of p38 in KF compared with NF. It did not affect the protein level of p38 in both KF and NF. Figure is representative of three experiments with similar results (normalized to NF group or without electronic beam treated group). Error bars in A-F indicate s.d.

The expression of Col-1 was mediated by p38 activation and the TGF-beta signaling pathway (Lam et al., 2005; Shih and Bayat, 2010). In this study, we first determined the expression of mir-21 and smad7 in KF and NF tissues. Results showed that mir-21 expression was downregulated while smad7 expression was upregulated in KF tissues compared with NF tissues (Fig. 3A), and an inverse correlation between mir-21 and smad7 expression was observed (Fig. 3B). To investigate the regulation of smad7 by mir-21, the luciferase activity test was used to determine if smad7 was a target of mir-21 using smad7 3′UTR. Our data showed that mir-21 only reduced luciferase activity in cells containing wild-type 3′ UTR, but not in cells containing mutant 3′UTR (Fig. 3C); and the expression level of mir-21 was significantly higher in KF compared with NF (*P*<0.05) (Fig. 3D). Next, we checked the effects of electron beam irradiation on the mRNA level of mir-21 with qPCR. As Fig. 3E indicates, the mRNA level of mir-21 in KF was decreased after electron beam irradiation in comparison with NF. However, electron beam irradiation significantly decreased the phosphorylation of p38 without any effects on the protein and mRNA levels of p38. Interestingly, the mRNA level of smad7 in KF was upregulated after electron beam irradiation compared with NF (Fig. 3F). Results from western blotting indicated that electron beam irradiation increased the expression of smad7 and suppressed the phosphorylation of p38 in KF compared with NF. It did not affect the protein level of p38 in both KF and NF (Fig. 3G). In summary, these observations elucidated that mir-21 was a direct controller of smad7 and that electron beam irradiation decreased the phosphorylation of p38 and modulated the expression of mir-21/smad7 signaling.

### Mir-p21 modulates the phosphorylation of p38 by regulating the protein level of smad7

**Fig. 4.**
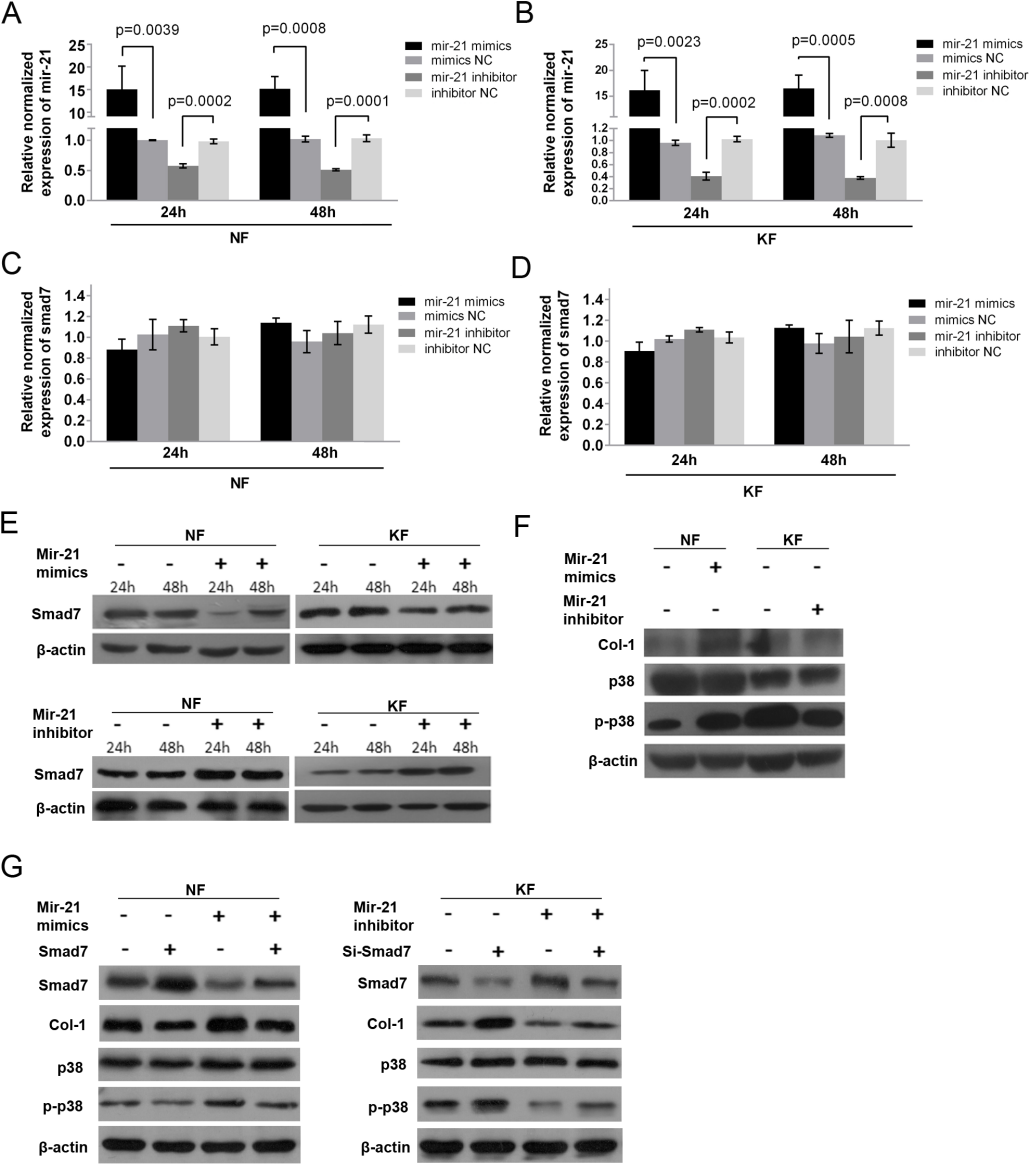
**Mir-21 modulates the phosphorylation of p38 by regulating smad7 at the protein level.** (A,B) qPCR data revealed expression levels of mir-21 in NF (A) and KF (B) were elevated with mir-21 mimics or inhibitor transfection after 24 and 48 h. (C,D) qPCR results indicated that there were no significant changes in the mRNA level of smad7 after mir-21 mimics or inhibitor transfection in NF and KF. (E) Western blots indicated that mir-21 mimic transfection decreased the protein level of smad7 both in NF and KF; however, the transfection of mir-21 inhibitor improved the protein level of smad7 in KF and NF. (F) Western blots showed that mir-21 mimic transfection increased the protein level of Col-1 and phosphorylation of p38 in NF without changing the expression of p38. (G) Western blot showed that overexpression of smad7 in NF partly restored the regulatory effect of mir-21 mimics on smad7, Col-1 and p-p38 expression; while suppression of smad7 by siRNA also partly restored the regulation effect of mir-21 inhibitor on those protein expression. Figure is representative of three experiments with similar results (normalized to mimics NC or inhibitor NC). Error bars in A-D indicate s.d.

To elucidate whether electron beam irradiation suppressed the expression of Col-1 by modulating mir-21/smad7 signaling, we transfected NF and KF with mir-21 mimics or inhibitors. Afterwards, the expression of smad7, p38 activation and the expression of Col-1 were detected with qPCR and western blotting. As Fig. 4A and B indicate, mir-21 mimic transfection increased the gene level in NF more than 15 times at 24 and 48 h after transfection, and mir-21 inhibitors transfection decreased the gene level in KF by more than 50% at 24 and 48 h. Interestingly, the overexpression or knockdown of mir-21 regulated smad7 at the protein level rather than the mRNA level (Fig. 4C-E). These data did not show any significant differences for the expression of any gene between the time points of 24 and 48 h. Therefore, we chose the time point of 48 h to detect Col-1 expression and p38 phosphorylation. As shown in Fig. 4F, after mir-21 mimic transfection, the Col-1 expression and p38 phosphorylation were both promoted in NF; however, the expression of p38 was not affected. After transfection with mir-21 inhibitors, Col-1 expression and p38 phosphorylation both declined in KF, while the expression of p38 was unaffected. Moreover, results from the western blot assay showed that overexpression of smad7 in NF partly restored the regulatory effect of mir-21 mimics on smad7, Col-1 and p-p38 expression; and suppression of smad7 by siRNA also partly restored the regulation effect of mir-21 inhibitor on the protein expression (Fig. 4G). These data clearly demonstrated that mir-21 negatively regulated the expression of smad7 to further control p38 activation and affect Col-1 synthesis.

In summary, all the data from Figs 2, 3 and 4 show that electron beam irradiation decreased Col-1 protein synthesis in KF through the mediation of mir-21/smad7/p38 signaling.

### Electronic beam irradiation mediated Col-1 synthesis via the mir-21/smad7 pathway in keloid-derived fibroblasts

**Fig. 5.**
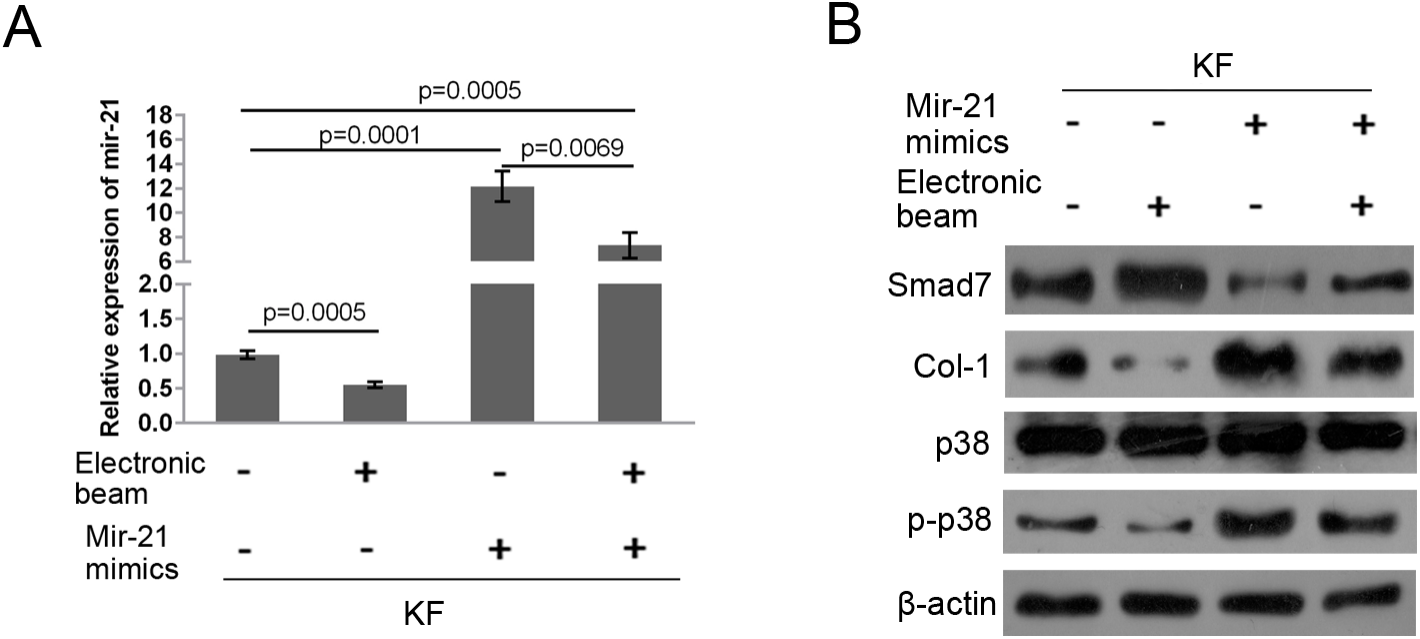
**Electronic beam irradiation mediated Col-1 synthesis via mir-21/smad7 pathway in keloid-derived fibroblasts.** (A) The expression level of mir-21 was downregulated by electronic beam irradiation; while mir-21 mimic transfection restored the inhibitory effect of electronic beam irradiation on mir-21 expression. (B) The expression levels of smad7 and p38 protein were promoted while the expression levels of Col-1 and p-p38 protein were reduced by the electronic beam irradiation; mir-21 mimic transfection could restore the regulatory effect of the electronic beam irradiation on protein expression of smad7, p38, Col-1 and p-p38. Figure is representative of three experiments with similar results (normalized to mimics NC or without electronic beam treated group). Error bars in A indicate s.d.

To further confirm that the electronic beam irradiation mediated Col-1 synthesis via mir-21/smad7 pathway in keloid-derived fibroblasts, mir-21 expression was determined in mir-21 mimic transfected keloid-derived fibroblasts under electronic beam irradiation. Results showed that electronic beam irradiation could significantly reduce the expression level of mir-21, while mir-21 mimic transfection could restore the inhibitory effect of the electronic beam irradiation on mir-21 expression (Fig. 5A). Moreover, the expression levels of smad7 and p38 protein were promoted while the expression levels of Col-1 and p-p38 protein were reduced by the electronic beam irradiation; mir-21 mimic transfection could restore the regulatory effect of the electronic beam irradiation on protein expression of smad7, p38, Col-1 and p-p38 (Fig. 5B). Taken together, these data suggested that the electronic beam irradiation mediated Col-1 synthesis via mir-21/smad7 pathway in keloid-derived fibroblasts.

## DISCUSSION

A keloid scar is characterized by exuberant fibro proliferation and excessive collagen deposition (Aarabi et al., 2007). It is not only aesthetically displeasing but can also be both painful and functionally disabling, which significantly impairs patients' quality of life (Bayat et al., 2003). Electron beam irradiation is commonly used with surgical removal to treat this disease because it is capable of suppressing reoccurrence. Although it has been reported that electron beam irradiation decreases collagen synthesis (Stadelmann et al., 1998), the underlying mechanism is unclear. This study is the first to show that electronic beam irradiation inhibits Col-1 expression at both the mRNA and protein levels by suppressing the expression of mir-21. Our data demonstrated that mir-21 directly targets smad7 to regulate its expression and the overexpression/knock down of mir-21, leading to p38 activation/deactivation. This implicated that smad7 may work as an upstream regulator of the p38 signaling pathway in keloids, which could be significantly downregulated with electron beam irradiation.

This was the first report indicating that mir-21 expression was inhibited by electron beam irradiation. As an important oncogenic miRNA, mir-21 is upregulated in several types of cancers, such as breast, lung, colon, pancreas, prostate and hematological cancers (Navarro et al., 2008; Iorio et al., 2005; Stefano et al., 2006). The overexpression of mir-21 increases cell proliferation, migration, invasion and metastasis in a number of cancer cell lines. Furthermore, mir-21 is overexpressed in fibrotic tissues, such as fibrotic lungs of patients with idiopathic pulmonary fibrosis and hypertrophic scarring (Chau et al., 2012; Liu et al., 2010). We also showed that mir-21 was highly expressed in KF by qPCR. The downregulation of mir-21 by electron beam irradiation severely damaged the proliferation rate and migration capacity of KF, especially Col-1 synthesis in KF. This information led to a better understanding of the relationship between mir-21 and Col-1. In this study, overexpression of mir-21 in NF with mir-21 mimic transfection resulted in the upregulation of Col-1 at mRNA and protein levels; this finding was also confirmed by the downregulation of mir-21 in KF upon transfection with mir-21 inhibitors.

Concurrently, smad7 was upregulated after electron beam irradiation. Smad7 was initially found to be an inhibitor of the TGF-β pathway (Yan et al., 2009), which is also downregulated in fibrotic tissues and is related to collagen synthesis. In addition to the TGF-β pathway, smad7 also could inhibit the bone morphogenetic protein (BMP) signal pathway which plays a crucial role in fibrosis and skin development (Moura et al., 2013; Zhang and Dressler, 2013). Therefore, the upregulation of smad7 may result in decreased collagen production (Tang et al., 2011). Our luciferase assay data revealed that smad7 was a direct target of mir-21, which was consistent with previous reports (Li et al., 2013). Therefore, mir-21 controlled Col-1 production via targeting smad7 expression. Our qPCR and western blot data showed that mir-21 regulated smad7 expression by suppressing protein translation rather than mRNA degradation. In addition, mir-21 was reported to modulate the BMP pathway effect by regulating BMP target genes (Ahmed et al., 2011), which may also contribute to the regulatory effect of mir-21 on Col-1 expression.

Furthermore, p38 phosphorylation was blocked by electron beam irradiation and depleted mir-21. The p38 signaling pathway played a balancing role between the synthesis of the extracellular matrix and degradation by matrix metalloproteinases (MMPs) (Lam et al., 2005). P38 could also be activated by the TGF-β pathway with smad7 as an adaptor (Li et al., 2014; Edlund et al., 2003; Iwai et al., 2008; Yan et al., 2009; Wang et al., 2013). We demonstrated that the regulatory effect of the electron beam irradiation could be partly restored by mir-21 overexpression achieved by mir-21 mimic transfection. Therefore, our data demonstrated that mir-21 controlled Col-1 expression by mediating the regulation of smad7 by p38 phosphorylation.

**Fig. 6.**
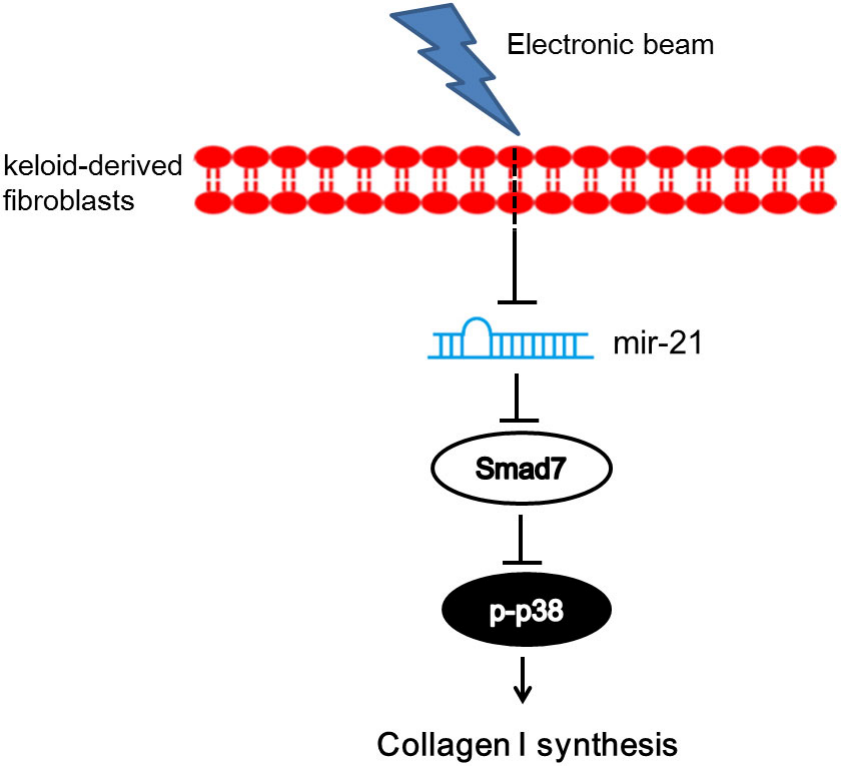
**A schematic diagram of the proposed mechanisms of electronic beam in Col-1 synthesis in keloid-derived fibroblasts.** The arrow indicates activation or induction, line with dead-end indicates inhibition or blockade.

In conclusion, mir-21/smad7/p38 signaling was involved in electron beam irradiation therapy. Via the above signaling pathway, electron beam irradiation therapy suppressed Col-1 expression at the mRNA and protein levels (Fig. 6). Our observations elucidated one possible mechanism for electron beam irradiation therapy inhibiting the recurrence of scars.

## MATERIALS AND METHODS

### Reagents and antibodies

Dulbecco's modified medium (DMEM) was purchased from Invitrogen (Carlsbad, CA, USA). Rabbit anti-human fibronectin, mouse anti-human p38 antibody, mouse anti-human phosphorylated p38 antibody and rabbit anti-human β-actin antibody were from Cell Signaling Technology (Danvers, MA, USA). The mouse anti-human Col-1 antibody and rabbit anti-human smad7 antibody were from Santa Cruz Biotechnology (Dallas, TX, USA). Goat anti-mouse IgG/HRP, goat anti-rabbit IgG/HRP and TRITC-labeled goat anti-rabbit IgG were from KPL Inc. (Gaithersburg, MD, USA). The pYr-MirTarget vector and pYr-MirTarget SMAD7-3′UTR (wild-type or mutant) were constructed by YRbio (Changsha, China). All primers for mir-21, Col-1, smad7, Fn, p38, U6 and GAPDH were synthesized by GenePharma (Shanghai, China).

### Primary keloid-derived fibroblast and normal skin fibroblast cultures

Six patients with keloid scarring used in this study completed written informed consent forms. This study was approved by the First People's Hospital of Chenzhou Committee on Biomedical Research Ethics. Keloid samples were obtained by surgical removal. Normal skin tissue was collected from locations at least 5 cm away from the keloid scars in each patient. Tissue samples were kept in DMEM supplemented with penicillin (100 U/ml) and streptomycin (50 µg/ml). Primary cell isolation was performed within 2 h after excision, as previously described (Arakawa et al., 1990). Briefly, keloid and normal skin tissues were cut into small pieces and seeded in cell culture flasks. Then, 72 h later, cells outgrew the explants culture. DMEM supplemented with 10% fetal bovine serum (FBS), penicillin (100 U/ml) and streptomycin (50 µg/ml) was used to maintain the cell culture and changed every three days. Subculture was performed when cells reached 80% confluence with a split ratio of 1:3.

### Electronic beam irradiation

KF and normal primary fibroblasts (NF) were planted in 6-well plates at a concentration of 5×10^5^/well. A 15 Gy dosage was applied to the cells at the top of the plates at 8 Mev with an electron beam irradiator (Siemens Primus) once. After a further 48 h culture, qPCR and western blot analysis were performed.

### Immunofluorescence staining

At passages 2-4, KF and NF were planted on coverslips and fixed with 4% paraformaldehyde. After being permeabilized with 0.3% Triton X-100, cells were incubated with fibronectin (Fn) and Col-1 primary antibodies for 2 h at room temperature. Then, a TRITC-labeled secondary antibody was added. Finally, coverslips were mounted on microscope slides and examined with an inverted fluorescent microscope.

### MTT assay

MTT assays were applied to measure the cell proliferation rates of KF and NF. Briefly, after electronic beam irradiation, cells were seeded in 96-well plates at a concentration of 5000 cells per well. Then, 72 h later, 10 µl of MTT (5 mg/ml) was added into each well and incubated for 4 h in cell incubators at 37°C without light. The cell medium was gently removed and DMSO was added to stop the reaction. Optical densities (OD) were measured with a microplate reader at 490 nm.

### Wound healing assay

After irradiation, one scratch was drawn with a 10 µl tip in each well of the 6-well plates. Pictures of the scratches were taken at time points of 0 h and 48 h with an inverted fluorescence microscope.

### MiRNA overexpression and knock down

The mir-21 mimics and inhibitor were purchased from GenePharma (Shanghai, China). The upregulation and downregulation of mir-21 was succeeded by transient mir-21 mimics and inhibitor transfection with Lipofectamine 2000 (Invitrogen, Carlsbad, CA, USA). All steps were completed according to the manufacturer's instructions. Briefly, cells were plated at 5×10^5^ per well in 6-well plates and cultured for 24 h. Then the cells were transfected with the mimics or inhibitors of mir-21 or negative control (NC) RNA at a final concentration of 50 nM, using Lipofectamine 2000 and serum-free Opti-MEM medium (GIBCO, Grand Island, NY, USA). After 6 h, the medium was replaced with DMEM with 10% FBS. qPCR evaluated the transfection efficacy at 24 h and 48 h after transfection.

### Smad7 overexpression and knock down

pCMV-inserted full-length human smad7 gene was purchased from Addgene (Cambridge, MA, USA). Specific small interfering RNA (siRNA) of smad7 were purchased from GenePharma (Shanghai, China). For smad7 overexpression treatment, cells (2×10^5^) grown on 6-well plates were transfected with 2 µg of pCMV-smad7 or pCMV using Lipofectamine 2000 and serum-free Opti-MEM medium. For smad7 knockdown treatment, cells (1.5×10^5^) grown on 6-well plates were transfected with 100 pmol siRNA of smad7 (si-Smad7) or negative control (si-NC) using 8 µl siRNA-Mate transfection reagent (GenePharma, Shanghai, China). After 6 h, the medium was replaced with DMEM with 10% FBS. The cells were harvested after 48 h. Western blot analyses were performed.

### Luciferase assay

The full-length 3′UTR of the human smad7 gene was amplified by PCR using TS-SMAD7-3′UTR (Yrbio, Changsha, China) as a template. The restriction site sequences for XhoI and NotI were added to the following primers: SMAD7-3′UTR–forward: CCGCTCGAGATCCTGTGTGTTAAGCTCT and SMAD7-3′UTR–reverse: AAAGCGGCCGCGGAGTCCTTTCTCTCTCAA. Site-directed mutagenesis was performed to generate smad7 3′UTR mutants containing mutations in the conserved mir-21 binding site using the following primers: SMAD7-3′UTR–Mutation Forward: ATGTTTAGACTTTAACTTATGCAATTTTTCTAACTA and SMAD7-3′UTR–Mutation reverse: TAGTTAGAAAAATTGCATAAGTTAAAGTCTAAACAT. PCR fragments were cloned into the XhoI site downstream of the luciferase gene in the pYr-MirTarget vector. The 293T cells were seeded at a concentration of 1×10^5^ cells in 24-well plates. The cells were co-transfected with 0.5 µg of pYr-MirTarget-SMAD7-3′UTR (wild type or mutant), 50 nM of mir-21 mimics, and a *Renilla* plasmid using Lipofectamine 2000, with empty vectors as controls. The cells were harvested 48 h later and assayed using the Dual-Luciferase Reporter Assay System and a multichannel microplate reader. The firefly luciferase values were normalized to the *Renilla* luciferase values expressed from the same pYr-MirTarget vector.

### qPCR

RNA was extracted from cells using the TRIzol Reagent. Cellular RNA was used for cDNA synthesis. For mir-21 qPCR, the total RNA was reverse transcribed with a miRNA-specific primer using the miScript Reverse Transcription kit (Qiagen, Hilden, Germany). For mRNA qPCR, total RNA was reverse transcribed with the Superscript Reverse Transcription kit (Thermo Fisher, MA, USA). Quantitative real-time PCR was performed using the SYBR Green Master Mix (Bio-Rad, CA, USA). The following primers were used: Col-1-forward: 5′-ATTGCCTTTGATTGCTGGGCAGAC-3′, Col-1-reverse: 5′-CAATGCTGCCCTTTCTGCTCCTTT-3′; Fn-forward: 5′-GACAGAGTTGCCCACGGTAA-3′, Fn-reverse: 5′-AGGAAAAAGACAGGACAAGAAGC-3′; Smad7-forward: 5′-CGATGGATTTTCTCAAACCAA-3′, Smad7-reverse: 5′-ATTCGTTCCCCCTGTTTCA-3′; p38-forward: 5′-TTCGCATGAATGATGGACTGAA-3′; p38-reverse:5′-GAACAAGACAATCTGGGAGGTG-3′; mir-21-forward: 5′-TTTTGTTTTGCTTGGGAGGA-3′, mir-21-reverse: 5′-AGCAGACAGTCAGGCAGGAT-3′; GAPDH-forward: 5′-CCAGGTGGTCTCCTCTGA-3′, GAPDH-reverse: 5′-GCTGTAGCCAAATCGTTGT-3′; U6-forward: 5′-CTCGCTTCGGCAGCACA-3′, U6-reverse: 5′-AACGCTTCACGAATTTGCGT-3′. The mRNA expression values were normalized to GAPDH. The miRNA expression values were normalized to U6. Relative expression levels of miRNA or mRNA were analyzed using the Bio-Rad C1000 Thermal Cycler.

### Western blot

Cells were decomposed with a protein lysis solution [50 mM Tris–HCl (pH 7.5), 150 mM NaCl, 1% SDS, 0.5% sodium deoxycholate and 0.5% Triton X-100] at a low temperature (4°C). Protease and phosphorylase inhibitors were added into the cellular lysate. The bicinchoninic acid assay was used to measure protein concentration. Western blot analysis was performed as previously described (Deng et al., 2015) using the following antibodies: mouse anti-human Col-1 antibody, rabbit anti-human smad7 antibody, mouse anti-human p38 antibody, mouse anti-human phosphorylated p38 antibody and rabbit anti-human β-actin antibody. Equal amounts of protein were resolved on 10% SDS-polyacrylamide gels and transferred to PVDF membranes. Horseradish peroxidase-conjugated goat anti-rabbit or mouse IgG was used as a secondary antibody. Bound fragments were detected with the ECL chemiluminescent kit (Pierce, Rockford, IL, USA) and exposed on X-ray film. Quantitative analysis of the protein band intensity by western blotting was performed using ImageJ software (National Institutes of Health, USA, https://imagej.nih.gov/ij/) and normalized to β-actin.

### Statistical analysis

Student's *t*-test was used for comparison between two groups. A *P*<0.05 value was considered statistically significant. Each test for independent experiments was repeated three times.

## References

[BIO018770C1] Aarabi S., Longaker M. T. and Gurtner G. C. (2007). Hypertrophic Scar formation following burns and trauma: new approaches to treatment. *PLoS Med.* 4, e234 10.1371/journal.pmed.0040234 17803351 PMC1961631

[BIO018770C2] Ahmed M. I., Mardaryev A. N., Lewis C. J., Sharov A. A. and Botchkareva N. V. (2011). MicroRNA-21 is an important downstream component of BMP signalling in epidermal keratinocytes. *J. Cell Sci.* 124, 3399-3404. 10.1242/jcs.086710 21984808 PMC3196856

[BIO018770C3] Arakawa M., Hatamochi A., Takeda K. and Ueki H. (1990). Increased collagen synthesis accompanying elevated m-RNA levels in cultured Werner's syndrome fibroblasts. *J. Invest. Dermatol.* 94, 187-190. 10.1111/1523-1747.ep12874489 2299193

[BIO018770C4] Bayat A., Mcgrouther D. A. and Ferguson M. W. (2003). Skin scarring. *BMJ* 326, 88-92. 10.1136/bmj.326.7380.88 12521975 PMC1125033

[BIO018770C5] Briones-Orta M. A., Tecalco-Cruz A. C., Sosa-Garrocho M., Caligaris C. and Macías-Silva M. (2011). Inhibitory Smad7: emerging roles in health and disease. *Curr. Mol. Pharmacol.* 4, 141-153. 10.2174/1874467211104020141 21222648

[BIO018770C6] Butler P. D., Longaker M. T. and Yang G. P. (2008). Current progress in keloid research and treatment. *J. Am. Coll. Surg.* 206, 731-741. 10.1016/j.jamcollsurg.2007.12.001 18387480

[BIO018770C7] Carthew R. W. (2006). Gene regulation by microRNAs. *Curr. Opin. Genet. Dev.* 16, 203-208. 10.1016/j.gde.2006.02.012 16503132

[BIO018770C8] Chau B. N., Xin C., Hartner J., Ren S., Castano A. P., Linn G., Li J., Tran P. T., Kaimal V., Huang X. et al. (2012). MicroRNA-21 promotes fibrosis of the kidney by silencing metabolic pathways. *Sci. Transl. Med.* 4, 397-400. 10.1126/scitranslmed.3003205 PMC3672221 22344686

[BIO018770C9] Deng S., Tang S., Zhang S., Zhang C., Wang C., Zhou Y., Dai C. and Xiao X. (2015). Furazolidone induces apoptosis through activating reactive oxygen species-dependent mitochondrial signaling pathway and suppressing PI3K/Akt signaling pathway in HepG2 cells. *Food Chem. Toxicol.* 75, 173-186. 10.1016/j.fct.2014.11.019 25434308

[BIO018770C10] Edlund S., Bu S., Schuster N., Aspenstrom P., Heuchel R., Heldin N.-E., ten Dijke P., Heldin C.-H. and Landstrom M. (2003). Transforming growth factor-beta1 (TGF-beta)-induced apoptosis of prostate cancer cells involves Smad7-dependent activation of p38 by TGF-beta-activated kinase 1 and mitogen-activated protein kinase kinase 3. *Mol. Biol. Cell* 14, 529-544. 10.1091/mbc.02-03-0037 12589052 PMC149990

[BIO018770C11] Iorio M. V., Ferracin M., Liu C.-G., Veronese A., Spizzo R., Sabbioni S., Magri E., Pedriali M., Fabbri M., Campiglio M. et al. (2005). MicroRNA gene expression deregulation in human breast cancer. *Cancer Res.* 65, 7065-7070. 10.1158/0008-5472.CAN-05-1783 16103053

[BIO018770C12] Iwai T., Murai J., Yoshikawa H. and Tsumaki N. (2008). Smad7 Inhibits chondrocyte differentiation at multiple steps during endochondral bone formation and down-regulates p38 MAPK pathways. *J. Biol. Chem.* 283, 27154-27164. 10.1074/jbc.M801175200 18644788

[BIO018770C13] Kashiyama K., Mitsutake N., Matsuse M., Ogi T., Saenko V. A., Ujifuku K., Utani A., Hirano A. and Yamashita S. (2012). miR-196a downregulation increases the expression of type I and III collagens in keloid fibroblasts. *J. Invest. Dermatol.* 132, 1597-1604. 10.1038/jid.2012.22 22358059

[BIO018770C14] Lam E., Kilani R. T., Li Y., Tredget E. E. and Ghahary A. (2005). Stratifin-induced matrix metalloproteinase-1 in fibroblast is mediated by c-fos and p38 mitogen-activated protein kinase activation. *J. Invest. Dermatol.* 125, 230-238. 10.1111/j.0022-202X.2005.23765.x 16098031

[BIO018770C15] Li Q., Zhang D., Wang Y., Sun P., Hou X., Larner J., Xiong W. and Mi J. (2013). MiR-21/Smad 7 signaling determines TGF-b1-induced CAF formation. *Sci. Rep.* 3, 2038-2038 10.1038/srep02038 PMC3687228 23784029

[BIO018770C16] Li H.-X., Wang C. M., H-J W. and Chen J. (2014). Artesunate restraining MAPK passage by smad7 to resist pulmonary fibrosis. *Eur. Rev. Med. Pharmacol. Sci.* 18, 3199-3204. 10.1007/s11255-016-1232-0 25487928

[BIO018770C17] Liu G., Friggeri A., Yang Y., Milosevic J., Ding Q., Thannickal V. J., Kaminski N. and Abraham E. (2010). miR-21 mediates fibrogenic activation of pulmonary fibroblasts and lung fibrosis. *J. Exp. Med.* 207, 1589-1597. 10.1084/jem.20100035 20643828 PMC2916139

[BIO018770C18] Moura J., da Silva L., Cruz M. T. and Carvalho E. (2013). Molecular and cellular mechanisms of bone morphogenetic proteins and activins in the skin: potential benefits for wound healing. *Arch. Dermatol. Res.* 305, 557-569. 10.1007/s00403-013-1381-2 23800970

[BIO018770C19] Navarro A., Gaya A., Martinez A., Urbano-Ispizua A., Pons A., Balague O., Gel B., Abrisqueta P., Lopez-Guillermo A., Artells R. et al. (2008). MicroRNA expression profiling in classic Hodgkin lymphoma. *Blood* 111, 2825-2832. 10.1182/blood-2007-06-096784 18089852

[BIO018770C20] Ogawa R., Miyashita T., Hyakusoku H., Akaishi S., Kuribayashi S. and Tateno A. (2007). Postoperative radiation protocol for keloids and hypertrophic scars: statistical analysis of 370 sites followed for over 18 months. *Ann. Plast. Surg.* 59, 688-691. 10.1097/SAP.0b013e3180423b32 18046154

[BIO018770C21] Seifert O. and Mrowietz U. (2009). Keloid scarring: bench and bedside. *Arch. Dermatol. Res.* 301, 259-272. 10.1007/s00403-009-0952-8 19360429

[BIO018770C22] Shih B. and Bayat A. (2010). Genetics of keloid scarring. *Arch. Dermatol. Res.* 302, 319-339. 10.1007/s00403-009-1014-y 20130896

[BIO018770C23] Stadelmann W. K., Digenis A. G. and Tobin G. R. (1998). Physiology and healing dynamics of chronic cutaneous wounds. *Am. J. Surg.* 176, 26S-38S. 10.1016/S0002-9610(98)00183-4 9777970

[BIO018770C24] Stefano V., Calin G. A., Chang-Gong L., Stefan A., Amelia C., Fabio P., Rosa V., Marilena I., Claudia R. and Manuela F. (2006). A microRNA expression signature of human solid tumors defines cancer gene targets. *Proc. Natl. Acad. Sci. USA* 103, 2257-2261. 10.1073/pnas.0510565103 16461460 PMC1413718

[BIO018770C25] Suh E. J., Remillard M. Y., Legesse-Miller A., Johnson E. L., Lemons J. M. S., Chapman T. R., Forman J. J., Kojima M., Silberman E. S. and Coller H. A. (2012). A microRNA network regulates proliferative timing and extracellular matrix synthesis during cellular quiescence in fibroblasts. *Genome Biol.* 13, R121 10.1186/gb-2012-13-12-r121 23259597 PMC3924601

[BIO018770C26] Tang B., Zhu B., Liang Y., Bi L., Hu Z., Chen B., Zhang K. and Zhu J. (2011). Asiaticoside suppresses collagen expression and TGF-β/Smad signaling through inducing Smad7 and inhibiting TGF-βRI and TGF-βRII in keloid fibroblasts. *Arch. Dermatol. Res.* 303, 563-572. 10.1007/s00403-010-1114-8 21240513

[BIO018770C27] Wang X., Li X., Ye L., Chen W. and Yu X. (2013). Smad7 inhibits TGF-β1-induced MCP-1 upregulation through a MAPK/p38 pathway in rat peritoneal mesothelial cells. *Int. Urol. Nephrol.* 45, 899-907. 10.1007/s11255-012-0350-6 23242502

[BIO018770C28] Wu Z.-Y., Lu L., Liang J., Guo X.-R., Zhang P. H. and Luo S.-J. (2014). Keloid microRNA expression analysis and the influence of miR-199a-5p on the proliferation of keloid fibroblasts. *Genet. Mol. Res.* 13, 2727-2738. 10.4238/2014.April.14.2 24782087

[BIO018770C29] Yan X., Liu Z. and Chen Y. (2009). Regulation of TGF-β signaling by Smad7. *Acta Biochim. Biophys. Sin.* 41, 263-272. 10.1093/abbs/gmp018 19352540 PMC7110000

[BIO018770C30] Yu H., Bock O., Bayat A., Ferguson M. W. J. and Mrowietz U. (2006). Decreased expression of inhibitory SMAD6 and SMAD7 in keloid scarring. *J. Plast. Reconstr. Aesthetic Surg.* 59, 221-229. 10.1016/j.bjps.2005.06.010 16676428

[BIO018770C31] Zhang P. and Dressler G. R. (2013). The Groucho Protein Grg4 suppresses Smad7 to activate BMP signaling. *Biochem. Biophys. Res. Commun.* 440, 454-459. 10.1016/j.bbrc.2013.09.128 24099773 PMC3947529

[BIO018770C32] Zhu H.-Y., Li C., Bai W.-D., Su L.-L., Liu J.-Q., Li Y., Shi J.-H., Cai W.-X., Bai X.-Z., Jia Y.-H. et al. (2014). MicroRNA-21 regulates hTERT via PTEN in hypertrophic scar fibroblasts. *PLoS ONE* 9, e97114 10.1371/journal.pone.0097114 24817011 PMC4016251

